# The molecular basis of herpes simplex virus latency

**DOI:** 10.1111/j.1574-6976.2011.00320.x

**Published:** 2012-01-10

**Authors:** Michael P Nicoll, João T Proença, Stacey Efstathiou

**Affiliations:** Division of Virology, Department of Pathology, University of CambridgeCambridge, UK

**Keywords:** herpesvirus, pathogenesis, miRNA, epigenetics, chromatin, reactivation, neurotropism

## Abstract

Herpes simplex virus type 1 is a neurotropic herpesvirus that establishes latency within sensory neurones. Following primary infection, the virus replicates productively within mucosal epithelial cells and enters sensory neurones via nerve termini. The virus is then transported to neuronal cell bodies where latency can be established. Periodically, the virus can reactivate to resume its normal lytic cycle gene expression programme and result in the generation of new virus progeny that are transported axonally back to the periphery. The ability to establish lifelong latency within the host and to periodically reactivate to facilitate dissemination is central to the survival strategy of this virus. Although incompletely understood, this review will focus on the mechanisms involved in the regulation of latency that centre on the functions of the virus-encoded latency-associated transcripts (LATs), epigenetic regulation of the latent virus genome and the molecular events that precipitate reactivation.

This review considers current knowledge and hypotheses relating to the mechanisms involved in the establishment, maintenance and reactivation herpes simplex virus latency.

## Introduction

Herpes simplex virus type 1 (HSV-1) and type 2 (HSV-2) are closely related ancient human pathogens responsible for a number of diseases of minor, moderate and severe pathology including oral and genital ulceration, virally induced blindness, viral encephalitis and disseminated infection of neonates. HSV-1 is typically transmitted during childhood, whilst HSV-2 is the cause of most genital herpes (Wald & Corey, 2007). Worldwide, the global prevalence of HSV-1 is approximately 90% with a prevalence of approximately 65% in the USA (Xu *et al*., 2002) and 52–67% in northern Europe (Pebody *et al*., 2004). HSV-2 infections are less frequent than HSV-1 infections with a prevalence of 10–20% in the USA and Europe (Wald & Corey, 2007). The success of HSV can be attributed to the establishment of lifelong persistent infection of the host, termed latency. During latent infection, no infectious virus is produced from infected cells, disease is absent from the host and transmission does not occur. This seemingly benign strategy is of critical importance to the survival strategy of the virus as infected cells maintained throughout the life of the host provide a reservoir for periodic reactivation – a process in which the virus re-enters the lytic replication programme.

HSV is a neurotropic virus and it is within sensory neurons that latent infection is established. Upon primary HSV replication at the oral or genital mucosa, the virus is able to infect the neuronal dendrites of sensory ganglia that innervate these tissues, and after retrograde microtubule-associated transport to the nerve cell body, the virus encounters a ‘choice' of gene expression programmes that dictates the fate of the neuron: lytic replication or latent infection. The latent virus genome is stably retained within sensory neurones and is characterized by repression of all viral lytic genes. In response to a variety of diverse stimuli, the virus can periodically reactivate to resume virus replication and produce infectious virus, which is transported anterograde back to the periphery to facilitate epithelial cell infection and consequent transmission.

## Models for the study of HSV-1 latency and reactivation

Although humans are the natural host of HSV, many, but not all, aspects of pathogenesis and latency can be modelled in experimental animals (for a detailed consideration of this topic, the reader is referred to the following reviews; (Wagner & Bloom, 1997; Dasgupta & Benmohamed, 2011). The mouse represents the most commonly used animal model system, and following peripheral infection, there is localized replication in epithelial cells followed by axonal transport to innervating sensory ganglia. During the acute stage of disease between 3 and 10 days postinfection, infectious virus can be readily detected within sensory ganglia but this is rapidly cleared by the developing adaptive immune response resulting in the establishment of latency. Unlike the situation in humans, spontaneous reactivation and the development of recurrent lesions are rarely observed in mice (Hill *et al*., 1975; Feldman *et al*., 2002; Gebhardt & Halford, 2005). For this reason, most studies of reactivation rely on explantation and culture of latently infected ganglia or the induction of reactivation *in vivo*, for example, by transient induction of hyperthermia (Sawtell & Thompson, 1992), CD8^+^ T cell depletion (Freeman *et al*., 2007; Orr *et al*., 2007) or skin abrasion (Hill *et al*., 1980). The other commonly used animal model is the rabbit eye model which, following infection with certain strains of HSV-1, leads to periodic spontaneous reactivation and the detection of infectious virus in animal tears; a situation more analogous to the asymptomatic shedding of HSV that occurs in humans (Koelle & Wald, 2000; Mark *et al*., 2008). Efficient reactivation can also be induced in this model by iontophoresis of epinephrine adding to its utility (Kwon *et al*., 1981). The guinea pig represents the model of choice for studies of HSV-2 latency. In this model, reactivation occurs spontaneously resulting in the formation of readily scored lesions.

*In vitro* systems, although easier to work with and more controllable than *in vivo* models, rely on an ‘unnatural’ block in lytic cycle replication through the use of antiviral drugs or the use of replication defective mutants and are often criticized for being highly artificial systems. Nonetheless, certain aspects of latency and reactivation have been addressed in such systems. For example, Wilcox *et al*. (1990) developed a model involving the infection of primary neuronal cultures derived from dorsal root ganglia of embryonic rats. Cultures maintained in acyclovir for 7 days resulted in latency establishment, and virus could be reactivated using a variety of stimuli such as the removal of nerve growth factor (NGF). Latency can also be efficiently established in primary neuronal cultures with virus mutants deficient for immediate early (IE) gene expression or with mutants defective for virus DNA replication and infectious progeny release (Arthur *et al*., 2001; Terry-Allison *et al*., 2007). Using such mutants, no drugs are needed to control primary infection. By measuring promoter activation, reactivation has been reported in this system following NGF withdrawal, histone deacetylase inhibition and infected cell polypeptide 0 (ICP0) expression (Arthur *et al*., 2001; Terry-Allison *et al*., 2007). Latently, infected cultures have also been established from sensory ganglia derived from latently infected mice, and in this system, the most efficient triggers of reactivation are heat shock or treatment with dexamethasone (Halford *et al*., 1996). A nonneuronal tissue culture latency model has also been developed that uses recombinant viruses severely impaired for IE gene expression to infect various nonneuronal cell types including human fibroblasts (Preston *et al*., 1997; Samaniego *et al*., 1998; McMahon & Walsh, 2008). The viral genome establishes a tightly repressed quiescent state, and quiescent genomes are not responsive to many of the reactivation stimuli that can de-repress latent genomes maintained in neuronal cells and can only be efficiently reactivated by the supply of ICP0 in trans (Harris *et al*., 1989; Coleman *et al*., 2008; Ferenczy *et al*., 2011). In these different *in vitro* latency model systems, the viral genome is always maintained in a circular form as found *in vivo*, but only in the neuronal systems are latency-associated transcripts (LATs) expressed and can the virus be easily reactivated.

## The process of productive HSV-1 infection

In most cell types, HSV-1 is a highly cytolytic virus and successful productive infection requires the efficient co-ordination of a large complement of genes coding for a diverse array of structural and nonstructural components. Expression of the HSV-1 lytic genes occurs in a temporal cascade that begins with immediate early (IE or α) genes and then proceeds through early (E or β), ‘leaky-late’ (L_1_ or γ-1), DNA replication and finally late (L_2_ or γ-2) gene expression (Honess & Roizman, 1974). Successful lytic replication is dependent on the expression of the viral IE genes within all infected cells. The application of protein synthesis inhibitors during infection of cell culture reveals the accumulation of six mRNAs, those corresponding to ICP0, ICP4, ICP22, ICP27, ICP47 and Us1.5, the IE genes (Honess & Roizman, 1974; Roizman *et al*., 2007). These genes are further defined by the presence of the TAATGARAT VP16-response elements (VRE) within their cognate promoters. VP16 is the main transcriptional activator of IE genes and is a late, structural component of the virus tegument layer, a complex network of proteins, which resides between the viral envelope and capsid (Wysocka & Herr, 2003; Roizman *et al*., 2007). Upon fusion and de-envelopment of the infecting virus, VP16 is released into the cytoplasm. The viral capsid is then transported to the nuclear membrane along the microtubule network and docks to the nuclear pore facilitating the release of viral DNA into the nucleus. Within the cytoplasm, VP16 is able to bind host cell factor-1 (HCF-1) protein that contains a nuclear localization sequence, and as a result of this association, VP16 is transported to the nucleus (Boissiere *et al*., 1999). Within the nucleus, the two proteins form a trimeric complex with the homeodomain protein Octamer binding protein-1 (Oct-1) on the consensus sequence VREs present in each IE promoter (Wysocka & Herr, 2003; Kristie, 2007). The potent VP16 activation domain in conjunction with the essential co-activator functions provided by HCF-1 then stimulates IE transcription and the HSV-1 lytic gene cascade is initiated. VP16 is however not essential for IE gene expression as mutants lacking the VP16 transactivation function can enter the lytic cycle, although with much reduced efficiency, particularly following low multiplicity infection. Hence, VP16 serves to increase the specific infectivity of virus DNA by enhancing IE gene transcription. This model of VP16 activation of IE gene expression is well understood (O'Hare, 1993; Wysocka & Herr, 2003; Kristie, 2007) but models in which it may fail are implicated and debated with regard to the establishment of neuronal latency. As such, these will be discussed in detail later in this review.

Following successful interaction of the VP16/Oct-1/HCF complex with virus DNA, the activation and accumulation of the HSV IE proteins serve to drive the transcription of viral genes whilst subverting host gene expression. Indeed, with the exception of ICP47 (an major histocompatability complex (MHC) class 1 evasion gene), all of the IE proteins are involved in gene regulation. Two of these proteins, ICP0 and ICP4, are both major transactivators of gene expression. ICP0 does not bind DNA and is a promiscuous transactivator of gene expression (Everett, 1984, 2000; O'Hare & Hayward, 1985; Cai & Schaffer, 1992). Although ICP0 is not essential for virus replication, HSV-1 mutants lacking ICP0 are driven towards a quiescent infection, resembling latency, following low multiplicities of infection (Everett *et al*., 2004). ICP0 is a multifunctional protein that associates with a large number of cellular proteins involved in transcription, cell cycle regulation, DNA repair and the interferon response (Everett, 2000; Hagglund & Roizman, 2004). ICP0 harbours an E3 ubiquitin ligase RING finger domain (Everett, 2000) and has been demonstrated to degrade cellular proteins including constituents of nuclear ND10 bodies; structures that otherwise restrict HSV infection in the absence of ICP0 (Everett, 1984; Van Sant *et al*., 2001; Boutell *et al*., 2002; Cuchet *et al*., 2011). ICP0 also disrupts the CoREST/REST repressor complex leading to dissociation of HDACs1/2 (Gu *et al*., 2005) and interacts with HDACs 4, 5 and 7 (Lomonte *et al*., 2004) implying an important role for ICP0 in antagonizing innate cellular repression of incoming viral genomes including histone-mediated gene silencing at the earliest stages of infection (Everett, 2000; Hagglund & Roizman, 2004; Everett *et al*., 2006). Available evidence is therefore supportive of the view that a major function of ICP0 is to create an environment that is favourable for virus transcription by directing the destruction of cellular repressors. These observations in addition to the ability of ICP0 to de-repress tightly silenced genomes in tissue culture models of quiescence (Harris *et al*., 1989; Stow & Stow, 1989; Preston *et al*., 1997; Samaniego *et al*., 1998; Coleman *et al*., 2008; Ferenczy *et al*., 2011) has implicated ICP0 in the earliest events of genome de-repression and has led to the attractive hypothesis that this protein could play a key role in the initiation of reactivation from latency.

Further IE genes provide numerous and important functions involved in promoting lytic infection and evading host immunity. Such functions include the inhibition of host mRNA translation by disruption of the RNA splicing machinery and promoting nuclear export of viral mRNAs by ICP27 (reviewed in Smith *et al*., 2005) and evasion of CD8^+^ T cell recognition via ICP47-mediated TAP inhibition (Früh *et al*., 1995; Hill *et al*., 1995). Collectively, the ‘purpose’ of the IE genes is to develop an active and robust environment in which to efficiently express the viral lytic programme. The collection of genes that comprise the early and late phases of replication can be broadly conceptualized as enzymes required for DNA replication and structural proteins required for assembly of infectious virus, respectively. These distinctions are necessarily concise and incomplete, but all together represent the classification of the > 80 genes encoded by the virus for productive replication.

## Virus transcription during latency

**Fig 1:**
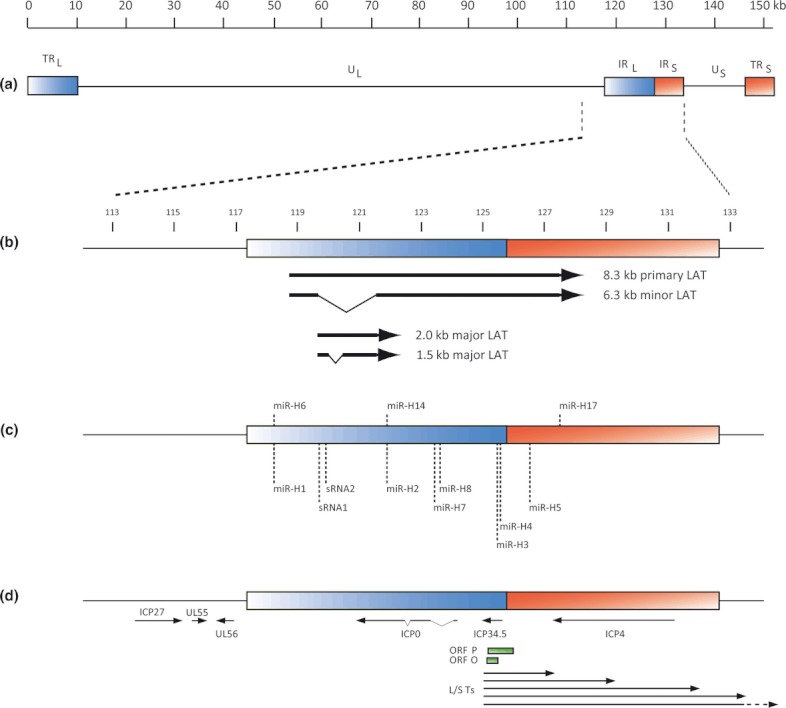
The genomic region of the HSV-1 LATs. (a) The prototypic organization of the HSV-1 genome. The 152 kb genome consists of the unique long (U_L_) and unique short (U_S_) sequences, flanked by inverted repeat sequences termed the terminal and internal long and short repeats (TR_L_, IR_L_, TR_S_ and IR_S_). (b) Enlarged view of the internal repeats displaying the coding location of the LAT RNA species. All LATs are processed from the 8.3 kb primary transcript, which is transcribed from a single promoter within the IR_L_. Above size markers represent kb. (c) Small noncoding RNAs expressed from within the LATs. With the exception of miR-H1, H6, H14 and H17, all displayed microRNA and sRNA are likely to be processed from the primary LAT transcript. (d) Lytic cycle virus genes encoded adjacent to or overlapping the LAT region. The L/ST transcripts represent a family of transcripts that are expressed late during productive infection from HSV-1 mutants lacking functional ICP4 (Yeh & Schaffer, 1993). Note that all genes displayed within the repeat sequences in b–d are diploid.

In stark contrast to the lytic programme, the only transcripts readily detectable during latency are the LATs (Fig. 1) (Wagner & Bloom, 1997). LATs are a set of co-linear RNAs transcribed from a locus within the repeat regions flanking the unique long region of the viral genome (Stevens *et al*., 1987). Their transcription leads to the production of an 8.3 kb ‘minor LAT’ primary transcript, which is then spliced to produce an unusually stable 2.0 kb intron which is further spliced to produce an additional stable 1.5 kb intron (Zabolotny *et al*., 1997). Together, these two RNA species are termed the ‘major LATs’. The nomenclature of these RNAs reflects their abundance and ease of detection within experimental systems. The inefficiently debranched stable lariat structure of the major LATs produced during splicing probably explains their abundance (Farrell *et al*., 1991; Wu *et al*., 1996, 1998; Krummenacher *et al*., 1997; Rødahl & Haarr, 1997). In contrast, the 8.3 kb minor LAT transcript and its 6.3 kb exon are difficult to reliably detect without more sensitive *in situ* hybridization (ISH) or RT-PCR methods (Rock *et al*., 1987; Deatly *et al*., 1988; Zwaagstra *et al*., 1990; Devi-Rao *et al*., 1991; Arthur *et al*., 1993). The scarcity of these RNAs may potentially reflect a low level of expression or/and rapid processing within the nucleus, but the latter theory is more likely. A number of recent studies have identified the presence of primary microRNA (miRNA) sequences throughout the HSV-1 and HSV-2 genomes, with the vast majority localized to the LAT locus (Cui *et al*., 2006; Tang *et al*., 2008, 2009; Umbach *et al*., 2008, 2009, 2010; Jurak *et al*., 2010). Excision of six such sequences from the 6.3 kb LAT exon of HSV-1 likely contributes to the rapid turnover of minor LAT.

The primary research focus with regard to the LATs has aimed towards understanding their role during latent infection. To date, this is still an area of quite some debate, but via the use of various small animal and tissue culture models, gross phenotypes and mechanisms of action are becoming apparent.

### Functions of the LATs

For a gene under such scrutiny concerning latent infection, the first perhaps surprising observation is that the LATs are not essential for latency establishment, maintenance or reactivation (Javier *et al*., 1988; Sedarati *et al*., 1989; Steiner *et al*., 1989). However, the most consistent *in vivo* phenotype associated with LAT-negative virus mutants has been a reduction in the efficiency of virus reactivation. This suggests that LATs must play a direct role during reactivation, but this theory has since been challenged by careful analysis of the murine latent cell reservoir by single cell contextual analysis (CXA) of viral DNA (Thompson & Sawtell, 1997). This study demonstrated that the LAT-negative viruses employed only established latency in 1/3 as many neurons as wild-type (WT) infection and that this correlated with decreased recovery of virus from mouse trigeminal ganglia following hyperthermic stress (a reactivation stimulus). Furthermore, hyperthermic stress applied during acute infection with LAT-negative viruses could yield similar numbers of latently infected neurons as WT virus, and under these preconditions, recovery of reactivated virus was equivalent (Thompson & Sawtell, 1997). Whilst it is likely that different infection models and specific methodologies affect the efficiency of latency establishment and reactivation (Perng *et al*., 2001; our unpublished observations), these data do provide a compelling argument that observed deficits in reactivation of LAT deficient mutants are the result of ablation of a LAT-encoded function responsible for enhancing the efficiency of latency establishment. Despite this, other studies have observed poor reactivation competence of LAT-negative virus when latent virus DNA loads are comparable to WT virus both in mice (Leib *et al*., 1989; Hoshino *et al*., 2009) and rabbits (O'Neil *et al*., 2004), suggesting that LATs influence latency during multiple phases of infection. However, with the appreciation that the latent virus DNA copy number within individual neurons can vary by greater than three orders of magnitude (Sawtell, 1997), whole ganglion quantification of virus DNA loads may not be an accurate reflection of the frequency of infected cells, an important variable that will be discussed later in this review.

How LATs actually mediate these gross effects *in vivo* is of clear importance to the strategy of persistence for HSV. A number of functions have been attributed to the LATs, but their mechanisms of action have not been forthcoming. Despite reports stating otherwise (Thomas *et al*., 1999; Henderson *et al*., 2009; Jaber *et al*., 2009), it is not generally accepted that the LATs are translated to a functional protein *in vivo*. However, with the recent description of LAT-encoded miRNAs (Fig. 1) (Cui *et al*., 2006; Tang *et al*., 2008, 2009; Umbach *et al*., 2008, 2009, 2010; Jurak *et al*., 2010), a bigger picture of the latent HSV transcriptome is becoming apparent.

### Repression of lytic gene expression

The expression of lytic cycle genes by the virus is detrimental to the progression of infection into latency. It would therefore be advantageous that expression of lytic transcripts could be curtailed by the virus in order to facilitate latency establishment. This idea necessitates the existence of an ‘active’ path into latency in a population of neurons, and experimental data suggest LATs may indeed exert this function. In 1997, two studies demonstrated the increased abundance of lytic gene transcripts during acute (Garber *et al*., 1997) and latent infection (Chen *et al*., 1997) of murine TG with a LAT deletion mutant. Although at low abundance, the levels of lytic cycle transcripts detected during latency were elevated by 5–10-fold with a LAT deletion mutant (Chen *et al*., 1997). It is tempting to speculate that a LAT-negative mutant could be at a greater propensity of spontaneous reactivation within murine TG, especially given the increased levels of TK mRNA, an early transcript dependent on the presence of ICP4 for expression (Chen *et al*., 1997). Subsequently, the production of mouse neuronal cell lines expressing various forms of the LAT locus continued to provide evidence that LAT could exert a repressive effect on IE mRNA levels (Mador *et al*., 1998). In the same study, the authors demonstrated that productive HSV replication was reduced in cell lines encoding the LAT locus during low multiplicity infection, but not in cell lines with a deletion in the promoter driving LAT transcription. However, this may not be the whole story. Giordani *et al*. (2008) have recently provided evidence that within a rabbit trigeminal ganglia model of infection, expression of the LATs leads to an enhanced accumulation of lytic transcripts during latency, directly conflicting conclusions drawn from investigations in the mouse. Further work will be required to ascertain the influence of different virus strains, routes of infection and specific animal model towards these conclusions, but the potential disagreement between animals is pertinent given their use in modelling human disease.

Currently, the majority of these aforementioned data support a role for the LATs in mediating the downregulation of genes required for lytic replication, but none of these studies provide a mechanistic explanation for this effect. It had been noted that the 2.0 kb LAT was encoded in the opposite orientation and overlapped the coding sequence of the virus IE gene ICP0, suggesting a potential regulatory role between maintenance and reactivation during latency. However, an antisense mechanism for the repression of ICP0 expression was discounted after investigation showed that provision of the 2.0 kb in *trans* failed to prevent accumulation of ICP0 mRNA or protein (Burton *et al*., 2003). Additionally, LAT sequences mapping to just the first 1.5 kb of minor LAT primary transcript are sufficient for efficient reactivation in animal models, and this region does not include the overlapping sequence between LAT and ICP0 (Perng *et al*., 1996).

Since the discovery of RNA interference roughly a decade ago, it has been swiftly recognized that DNA viruses encode the capacity to regulate gene expression via small noncoding RNAs (sRNAs). In particular, virus-encoded miRNAs are found in the majority of herpesviruses studied to date (Umbach & Cullen, 2009; Jurak *et al*., 2011) and have been implicated in the regulation of several key processes such as latent/lytic cycle control, immune evasion and cell survival (Boss *et al*., 2009). Not only do these regulatory sRNA genes raise the coding capacity of these viruses without large increases in genome length, but also by achieving their effects without coding for proteins, small RNA-based gene products remain intrinsically nonimmunogenic. This latter concept puts these molecules in a position of great value to viruses that rely on persistence and immune evasion to complete their infection cycles. HSV-1 and HSV-2 have been shown to encode 16 and 18 miRNAs, respectively, and there is a concentration of miRNAs within or adjacent to the LAT locus. In the case of HSV-1, six miRNAs (miR-H2, H3, H4, H5, H7 and H8) are encoded within the 8.3 kb primary LAT (Fig. 1). MiR-H1 and H6 are located just upstream of the LAT transcription start site, are abundant during productive infection and represent overlapping miRNAs with miR-H6 being transcribed in the opposite orientation to LAT (Cui *et al*., 2006; Umbach *et al*., 2008; Jurak *et al*., 2010). In addition to being expressed during productive infection, miRs H1 to H8 are expressed during latency. However, there is considerable variation in the expression levels of individual miRNAs during latency, with miRs H2, H3 and H4 being the most abundant as deduced by the frequency of sequence reads obtained from deep sequencing and/or quantitative stem-loop real-time reverse transcription PCR (Umbach *et al*., 2008, 2010; Jurak *et al*., 2010, 2011; Kramer *et al*., 2011). The targets of only four HSV-encoded miRNAs have been defined to date and are so far restricted to virus-encoded RNAs. In 2008, Umbach and colleagues were the first to demonstrate that HSV-encoded miRNAs were capable of downregulating the translation of viral genes. By co-transfecting plasmids over-expressing individual miRNA and their predicted protein targets, HSV-1 miR-H2 and miR-H6 were shown to reduce ICP0 and ICP4 protein abundance, respectively, whilst not affecting cognate mRNA levels (Umbach *et al*., 2008). Studies following this demonstrated that HSV-2 miR-H2 can inhibit the expression of ICP0 and HSV-2 miR-H3 and H4 can downregulate the expression of the neurovirulence factor ICP34.5 (Tang *et al*., 2008, 2009). LAT-encoded miRNAs have been detected during latency in animal models as well as analysis of postmortem human tissue (Tang *et al*., 2008, 2009; Umbach *et al*., 2008, 2009, 2010; Jurak *et al*., 2010) but their exact role in the regulation of the latency/reactivation cycle is not known. In the case of miRs targeting IE genes, a role in suppression of entry into the lytic cycle and therefore enhancement of latency establishment or maintenance is attractive and warrants further attention. It will be of particular interest to determine the impact of specific miRNA mutations/deletions on IE gene expression in the context of mutant virus infection both *in vitro* and *in vivo*. Defining the role of virus-encoded miRNAs in the natural history of HSV, infection will however prove challenging owing to the inherent limitations of the available animal model systems and a lack of information concerning potential cellular targets.

So do these studies relegate the LAT transcript to merely a large primary microRNA precursor? This is intriguingly not so. Indeed, the previously cited study by Mador *et al*. utilized cell lines that encoded, a region of LAT in which no miRNA proposed to target ICP0 or ICP4 is found. However, within this region, two sRNA species have recently been discovered (Peng *et al*., 2008). Both of these sRNAs were found capable of inhibiting productive infection in tissue culture, but to different extents. Despite showing only weak repression of virus replication by comparison with sRNA1, sRNA2 exhibited downregulation of ICP4 protein accumulation whilst not affecting cognate mRNA levels (Shen *et al*., 2009). At 62nt and 36nt length (sRNA1 and sRNA2, respectively), these RNAs do not appear to function as microRNA, although owing to the reduction in ICP4 protein levels in these experiments such an RNA interference mechanism may occur (Shen *et al*., 2009).

It is clear that small noncoding RNAs such as miRNA are likely to be central to the regulatory effort of the virus towards the regulation of lytic gene expression during latency or its establishment.

### Promotion of cell survival

Another major observation attributed to the expression of the LATs is the inhibition of cell death in response to virus infection. HSV-1 and HSV-2 encode a number of genes that interfere with the induction of apoptosis during lytic replication, including glycoproteins gD and gJ, as well as protein kinase U_s_3, ICP27 and ICP10 (Leopardi *et al*., 1997; Jerome *et al*., 1999; Zhou *et al*., 2000; Perkins *et al*., 2002). However, during latency, these gene products are presumably not expressed and therefore cannot counter triggers of apoptosis that may be encountered during latency establishment, maintenance and at the earliest stages of reactivation. A number of studies have attributed anti-apoptotic functions to the LATs. Perng *et al*. (2000) observed extensive apoptosis in rabbit trigeminal ganglia during the first 2 weeks of infection, utilizing TUNEL staining and antibody detection of caspase-3 cleaved poly (ADP-ribose) polymerase. Another alphaherpesvirus, bovine herpesvirus 1 (BHV-1), expresses a latency-related (LR) protein, which has a demonstrated anti-apoptosis function (Ciacci-Zanella *et al*., 1999; Henderson *et al*., 2004) and expression of this protein from HSV-1 deleted for LAT expression rescued the reactivation phenotype of this virus in rabbits and mice (Perng *et al*., 2002). Two further studies observed rescue of the LAT-negative reactivation phenotype utilizing the baculovirus anti-apoptosis gene product cpIAP or cellular FLICE-like inhibitory protein instead of LR BHV-1 protein (Jin *et al*., 2005, 2008). However, none of these three reports characterized the latent DNA loads during infection and so whether these viruses establish equivalent latency to WT virus is not clear. CXA of latently infected murine TG has shown that during LAT-negative virus infection, an increased loss of neurons occurs during establishment (Thompson & Sawtell, 2001). Additionally, both the absence (Thompson & Sawtell, 2001) and presence (Branco & Fraser, 2005) of apoptosis in murine TG have been reported. If anything is clear from these collective data, it is that the presence of a virally encoded product comprising or encoded within LAT is capable of supporting cell survival, and that this capacity is beneficial for the establishment and reactivation phenotypes of HSV. The mechanism by which this survival is mediated has been studied using various *in vitro* methodologies.

Inman *et al*. (2001) demonstrated that the sequences within the first 1.5 kb of LAT that are essential for efficient spontaneous reactivation in rabbits also map to regions of the LAT that promote survival of monkey kidney and mouse neuroblastoma cells following treatment with pro-apoptosis compounds such as etoposide and sodium butyrate. This work functionally linked the *in vitro* assessment of apoptosis inhibition and *in vivo* reactivation phenotype. This work was corroborated by a further study in HeLa and neuron-like SY5Y cells that mapped inhibition of caspase-8-mediated apoptosis to sequences within the LATs (Ahmed *et al*., 2002). Together, these studies agreed that anti-apoptosis activity of the LATs mapped to the 3′ end of the first exon, as well as in the 5′ end of the stable 2.0 kb LAT intron (Inman *et al*., 2001; Ahmed *et al*., 2002). These studies were furthered with the production of murine neuroblastoma cell lines expressing the first 3225 bp of LAT from the HSV latency associated promoter (LAP). These cell lines were more resistant to cold shock-induced apoptosis, leading to increased survival and reduced levels of DNA laddering compared to both WT cells and cells in which the LAP had been deleted from the LAT construct (Carpenter *et al*., 2007). Importantly, this study also demonstrated that cell lines with robust LAT expression were able to inhibit the cleavage (and thus activation) of caspase 3 in the absence of other virus genes. Others have also found that LAT expression promotes survival of primary neuronal cultures following NGF withdrawal from culture (Hamza *et al*., 2007).

Whilst these studies demonstrate a functional role in cell survival, a mechanism by which LAT could exact this outcome is still lacking. As previously described in this review, two sRNA species have been discovered within the 3′ end of the LAT exon (Peng *et al*., 2008). In a functional analysis, an increase in cell survival following cold shock could be observed in cells co-transfected with plasmids expressing both sRNA (Shen *et al*., 2009). These results appear not to be consistently replicated for each sRNA in isolation, but sRNA1 appears to mediate the majority of this activity as suggested via mutagenesis analysis (Shen *et al*., 2009). As previously described, the length of these RNAs indicates that they are not miRNA. Therefore, even if these RNAs were to represent *bona fide* effector molecules, the mechanistic action by which the LATs promote cell survival currently still eludes our understanding.

## Latency establishment

HSV-1 latency establishment in sensory neurons has long been thought to be the default path of a failure to initiate IE gene expression (reviewed in Preston, 2000; Efstathiou & Preston, 2005). Consistent with this view is the fact that virus mutants severely impaired for the initiation of lytic cycle, such as mutants in one or more IE genes or VP16 (Chiocca *et al*., 1990; Dobson *et al*., 1990; Katz *et al*., 1990; Steiner *et al*., 1990; Ecob-Prince *et al*., 1993; Sedarati *et al*., 1993; Marshall *et al*., 2000) are able to establish latent infection *in vivo*. Moreover WT virus can establish latent infection in sensory ganglia that do not directly innervate the site of primary infection and where no previous IE gene expression can be detected at any time prior to the establishment of latency (Speck & Simmons, 1992; Lachmann *et al*., 1999). It appears that viral DNA delivery into the neuronal nucleus is the only requisite for the establishment of latency.

As described earlier, efficient lytic cycle gene expression is dependent upon the transactivating function of the VP16-induced complex that is formed by the structural tegument protein VP16, HCF-1 and Oct-1 (reviewed by Wysocka & Herr, 2003). In sensory neurons, however, the formation of the VP16-induced complex is thought to be severely impaired owing to restrictions in the availability of all its members. It has been suggested that VP16 might not be efficiently transported along axons such that insufficient amounts reach the neuronal cell body (Kristie & Roizman, 1988; Kristie *et al*., 1999). HCF-1 has been shown to have a distinct cellular localization in sensory neurons, where it has been detected exclusively in the cytoplasm (Kolb & Kristie, 2008) and is therefore unavailable to participate in the formation of a VP16-induced complex. Oct-1 on the other hand has been shown to be downregulated in neuronal cells (Lakin *et al*., 1995). Other POU domain transcriptional factors are present in neurons, such as N-Oct2, N-Oct3, Brn-2, Brn-3a or Brn-3b and although these proteins can recognize the same sequences in IE promoters, they mostly have repressive effects (Lillycrop *et al*., 1991; Hagmann *et al*., 1995; Latchman, 1999). These restrictions for the formation of the VP16-induced complex and consequently inefficient IE promoter activation are in agreement with latency being the default path of an IE gene block; however, there is evidence to support the theory that there might be other paths leading to latency establishment.

Following infection of experimental animals, HSV-1 is able to go through an acute phase of infection in the ganglia that directly innervate the site of infection and there is an early divergence of the pathways leading to either productive or latent infection (Margolis *et al*., 1992; Speck & Simmons, 1992; Lachmann *et al*., 1999). Nonetheless, a small proportion of neurones (< 1%) expresses both viral antigen and LATs during acute infection (Margolis *et al*., 1992; Speck & Simmons, 1992) raising the possibility that expression of lytic cycle genes may not preclude entry into latency. It has also been shown that a subpopulation of latently infected neurons harbour high numbers of viral DNA molecules that can go up to thousands of genomes per cell (Efstathiou *et al*., 1986; Slobedman *et al*., 1994; Sawtell, 1997). If these neurons are a result of abortive lytic infection post-DNA replication or the result of high viral seeding from the periphery/ganglia is currently unclear.

Studies with HSV-1 mutants lacking a functional TK gene have shown that neurones containing high latent viral DNA loads could still be achieved even in the absence of TK, demonstrating that neurons *in vivo* have the potential to survive very high viral inputs transported from peripheral sites (Thompson & Sawtell, 2000; Wakim *et al*., 2008). However, the question still remains whether the neurons that receive high numbers of genomes would have survived infection with WT virus because TK mutants are severely compromised for replication in neurons.

One possible mechanism that could account for this high loading of viral DNA would be the noncytolytic CD8^+^ T cell inhibition of neuronal HSV-1 replication (Khanna *et al*., 2004; Decman *et al*., 2005). This mechanism is able to arrest HSV-1 reactivation in a noncytolytic fashion owing to a block in the viral gene expression cascade induced by IFN gamma secretion and the degradation of the ICP4 protein by CD8^+^ T cell-derived Granzyme B (Knickelbein *et al*., 2008) and could explain the CD8^+^ T cell-dependent termination of virus replication in the absence of neuronal destruction during the late stages of acute ganglionic infection (Simmons & Tscharke, 1992). The observation that immune infiltrates composed largely of CD8^+^ T cells is a feature of both latently infected murine and human trigeminal ganglia (Liu *et al*., 1996; Theil *et al*., 2003) is explained by the recognition of virus antigen on rare neurones undergoing spontaneous reactivation. CD8^+^ T cells are therefore likely to play an important role in the control of reactivation in a manner independent to LAT-encoded miRNAs suppression of IE gene expression (Held *et al*., 2011). Surprisingly, the CD8^+^ T cells involved in this mechanism in the C57BL/6 mouse model have been found to be specific for an immunodominant glycoprotein B (gB) epitope and not IE proteins. gB is a leaky-late gene and, the fact that the reactivating neuron was recognized by a gB-specific CD8^+^ T cell indicates that IE and early genes are likely to be expressed prior to a CD8^+^ T cell-mediated block in reactivation. These data indicate that, not only are neurons able to survive high viral genome inputs, they can also survive extensive viral gene expression (Kramer *et al*., 1998; Ramachandran *et al*., 2010). This is in agreement with studies using primary neuronal cultures where upon infection with replication defective mutants, transient IE promoter activity was detected in the majority of the neurons in culture prior to the establishment of latent infection (Arthur *et al*., 2001). A previous study by Wilcox and colleges revealed that primary neuronal cultures infection by an ICP0 ring domain mutant resulted in significantly less neurons expressing LATs in comparison with WT virus. In these experiments, the levels of LAT expression were found to correlate with viral DNA loads revealing a deficit in the establishment of latent infection by the ICP0 mutant. This effect could not be detected with an ICP4 mutant virus or when ICP0 was supplied in trans by an adenovirus vector (Wilcox *et al*., 1997). These data suggest that under certain circumstances, IE ICP0 expression may function to enhance latency establishment. A more recent study using Cre reporter animals and WT HSV-1 recombinants with Cre recombinase under control of the ICP0 promoter revealed that there is a subpopulation of latently infected cells that experience ICP0 promoter activation prior to latency establishment *in vivo* (Proença *et al*., 2008). A continuation of this study has extended the range of promoters tested and has revealed that ICP4 promoter activity is also compatible with latency establishment whilst TK and VP16 promoter activation is largely incompatible (Proença *et al*., 2011). These data indicate that a subpopulation of latently infected cells *in vivo* experience IE promoter activity prior to genome silencing and the establishment of neuronal latency. Of potential relevance, here is the observation that alternative spliced forms of ICP0 have been reported during productive infection (Everett *et al*., 1993; Carter & Roizman, 1996), at least one of which functions as a dominant negative repressor of ICP0-mediated transactivation in transient expression assays (Weber & Wigdahl, 1992; Weber *et al*., 1992). Whether the results obtained from reporter mouse model systems is indicative of IE protein expression being compatible with the establishment of latency is currently unclear; however, the results obtained with this model system are difficult to reconcile with a simple default model of latency establishment and argue for the operation of posttranscriptional mechanisms to restrict lytic cycle progression.

Studies with replication defective mutants have shown that the minimal requirement for latency establishment seems to be the delivery of viral DNA to the neuronal nucleus. However, as discussed, WT viruses may follow alternate pathways into latency. Therefore, latency can be established via mechanisms that do not involve a complete block in viral gene expression and might even rely on some viral proteins/transcripts in order to efficiently establish latent infection.

### The HSV genome and epigenetic regulation of gene expression

HSV-1 possesses a linear double-stranded DNA genome of 152 kb (reviewed by McGeoch *et al*., 2006). The virus genome is divided into two ‘unique’ regions (unique long and short or U_L_ and U_S_ regions) and each is flanked by inverted repeats (Fig. 1). Following nuclear entry, the linear genome circularizes before the advent of viral protein production (Garber *et al*., 1993; Strang & Stow, 2005). During lytic infection, early genome replication is achieved by a theta replication mechanism initiated at three redundant origins of replication (two copies of oriS and one copy of oriL) but rolling circle replication later predominates, forming ‘endless’ DNA lacking termini (reviewed by Boehmer & Nimonkar, 2003; Muylaert *et al*., 2011). This concatemeric DNA is later cleaved to monomeric units during packaging into newly formed capsids. In contrast to lytic infection, HSV DNA isolated from murine and human latently infected tissue demonstrates a lack of genomic termini, suggesting the HSV genome is maintained as a circular episome during latency (Rock & Fraser, 1983; Efstathiou *et al*., 1986) and the latent viral genome is assembled into nucleosomes (Deshmane & Fraser, 1989). In contrast to members of the lymphotropic gammaherpeviruses, that establish latency within dividing cell populations, the neuronal cell tropism of HSV latency negates the requirement for virus-encoded cis- and trans-acting genome maintenance functions.

**Fig 2:**
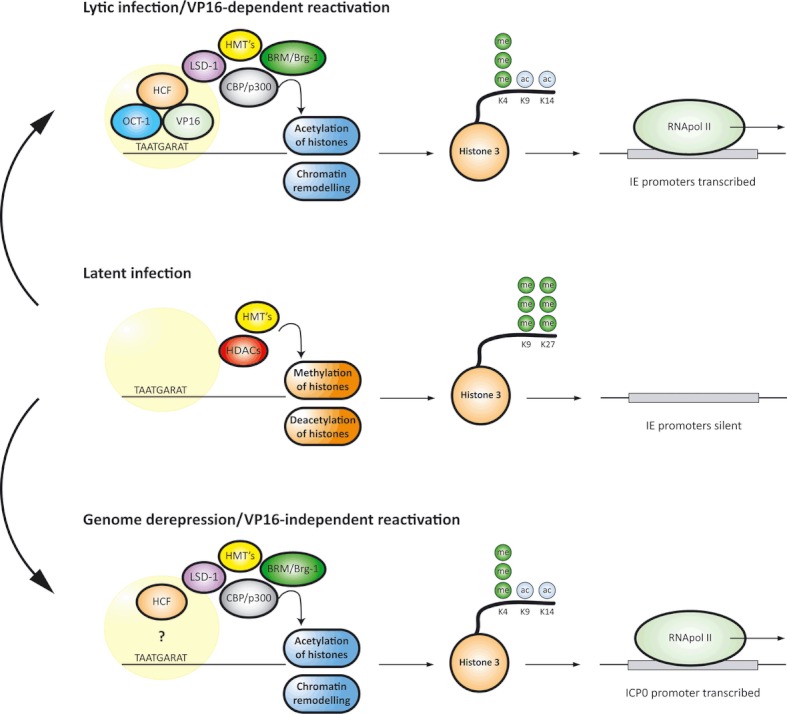
Chromatin control of HSV-1 gene expression. Two models of reactivation are shown, involving the specific activation of the virus regulatory proteins VP16 or ICP0 by cellular factors. Lytic infection/VP16-dependent reactivation: The VP16/Oct-1/HCF trimeric complex interacts with a number of co-activators including histone acetyltransferases (CBP/p300), chromatin remodelling factors (BGRF-1 and BRM), histone methyltransferases (HMT's) and lysine-specific demethylase-1 (LSD-1). Recognition of IE promoters by the VP16-induced complex results in activation of IE gene expression and prevention of repressive histone accumulation on the virus genome. Histone modifications associated with active transcription, such as H3K4me3 and H3K9ac, are associated with the virus genome during lytic infection. Latent infection: In the absence of virally encoded transactivators, HMT's and histone deacetylases (HDACs) maintain viral lytic promoters in a repressed chromatin state characterized with the accumulation of histone modifications associated with repression, such as H3K9me3, resulting in silencing of gene expression. An exception to this global silencing is the LAT locus, which is actively transcribed during latency. Genome depression/VP16-independent reactivation: In the absence of VP16, the presence of some or all cellular co-activators involved in the activation of IE promoters during lytic infection could facilitate gene expression following reversal of heterochromatin-based silencing. In this model, specific acetylation of the ICP0 promoter region would result in ICP0 gene expression, leading to further depression across the virus genome and reactivation.

During infection, HSV DNA entering the nucleus becomes rapidly associated with histones, in a cellular response presumably aimed at silencing foreign gene expression (Fig. 2). Whilst the role of this regulation on gene expression shall be briefly considered, readers are directed to recent, thorough reviews on chromatin control of HSV infection (Knipe & Cliffe, 2008; Kutluay & Triezenberg, 2009; Bloom *et al*., 2010; Nevels *et al*., 2011). In contrast to the lymphotropic gammsherpesviruses, the HSV genome does not appear to be methylated at CpG residues during latency; thus, this form of epigenetic regulation is presumably not utilized by the virus (Dressler *et al*., 1987; Kubat *et al*., 2004b). Instead, the association of histones consistent with partial or unstable nucleosomal structure (Lacasse & Schang, 2010) is believed to mediate epigenetic control of specific viral promoters. This control is thought to be regulated by posttranslational modification (PTMs) of histones occupying key lytic and latent viral promoters (Fig. 2). Indeed, during lytic infection, activating euchromatin-like modifications such as acetylation of H3K9 (histone H3, lysine 9 from the N-terminus) and H3K14 are enriched upon lytic gene promoters, whilst repressive heterochromatin-like modifications such as di-methylation of H3K9 are underrepresented (Herrera & Triezenberg, 2004; Kent *et al*., 2004; Huang *et al*., 2006). In direct contrast, during latency, the actively transcribed LAT locus undergoes enrichment of acetylated H3K9 and H3K14 at the LAT promoter and enhancer, with these modified histones not present at the ICP0 promoter or DNA polymerase gene (Kubat *et al*., 2004a, b). During latency, viral lytic genes are enriched with methylated H3K27 (a marker of facultative heterochromatin) as well as methylated H3K9 (a marker of constitutive heterochromatin) (Wang *et al*., 2005b), demonstrating that the latent HSV genome is associated with both reversible and nonreversible repressive chromatin modifications (Cliffe *et al*., 2009; Kwiatkowski *et al*., 2009). Induction of reactivation by the explantation of latently infected mouse dorsal root ganglia results in a decrease in LAT RNA and is associated with a decrease in association with acetylated H3K9/K14 on the LAT promoter and a concomitant increase in the association of these activating histone marks on the now transcriptionally active ICP0 promoter (Amelio *et al*., 2006). Studies of LAT mutant viruses in the mouse have revealed a reduction in the association of both constitutive and facultative chromatin with lytic gene promoters (Wang *et al*., 2005b; Cliffe *et al*., 2009) suggesting that LATs facilitate the assembly of heterochromatin on lytic cycle promoters during latency. The ability of LATs to help maintain the epigenetic silencing of lytic gene promoters is consistent with phenotypic analyses of LAT deficient mutants, which are associated with increased lytic gene expression during latency (Chen *et al*., 1997; Garber *et al*., 1997). In contrast to the situation in the mouse, studies in rabbits have led to an opposing view on the role of LATs in the regulation of lytic cycle promoter activity. Thus, in rabbits, LATs function to keep the virus genome in a more transcriptionally active state (Giordani *et al*., 2008). Furthermore, a recent report using a mouse model has indicated that LATs may function to suppress the accumulation of facultative heterochromatin on the latent virus genome (Kwiatkowski *et al*., 2009). At the present time, it is difficult to reconcile these fundamentally opposing views of the role of LATs in epigenetic regulation of the latent virus genome. Clearly, further studies are warranted to clarify the precise role of LATs in epigenetic control of the latent virus genome and in particular to what extent results can be influenced by virus strain and animal model differences.

## The role of the CoREST/REST repressor complex in the regulation of productive and latent infection

Recruitment of the lysine-specific demethylase LSD1 to IE promoters by HCF1 has been shown to be crucial for the initiation of IE transcription during both productive infection and reactivation from latency (Liang *et al*., 2009). LSD1 is normally found as a component of a cellular repressor complex that includes histone deacetylase 1 or 2 (HDAC1/2) and co-repressor of REST (CoREST). In this context, the LSD1 repressor complex is recruited to specific DNA elements by repressor element-1 silencing transcription (REST) and functions as a corepressor by demethylating histone H3K4 and functions to repress neuronal gene expression in nonneuronal cells. How then does LSD1 function to stimulate viral IE gene expression? The function of LSD1 is known to be context-dependent (Lee *et al*., 2005; Shi *et al*., 2005) and under certain circumstances and when dissociated from HDAC1/2 and CoREST can function as a co-activator by the demethylation of repressive H3K9 marks. Thus, recruitment of LSD1 to IE promoters by virtue of its interaction with HCF-1 antagonizes the accumulation of repressive methylated H3K9 marks at IE promoters to facilitate IE transcription. ICP0-mediated disruption of the CoREST/REST repressor complex also plays a role in the transition from IE to early gene expression. Thus, ICP0 has been shown to interact with CoREST and functions to disrupt the cellular corepressor complex, which includes CoREST, REST and HDAC1/2 (Gu *et al*., 2005; Gu & Roizman, 2007, 2009). Biologically, disruption of CoRest/REST complex by ICP0 is of importance because the expression of a dominant negative CoREST protein in place of ICP0 results in improved replication of an ICP0 mutant virus (Gu & Roizman, 2007) and decreased replication of a virus mutant containing a lesion within the CoREST interaction region of ICP0 has been observed (Gu & Roizman, 2009). However, whether CoREST is directly involved in repressing HSV-1 infection in the absence of ICP0 is less clear because depletion of CoREST by ShRNA did not improve the replication of an ICP0 mutant *in vitro* (Everett, 2010). During latency *in vivo*, it is also unclear whether CoREST plays a direct role in the repression of viral gene expression and hence the maintenance of latency. Although it has been shown that a virus recombinant encoding dominant negative REST incapable of recruiting CoREST or LSD1 is more virulent than WT or control viruses (Du *et al*., 2010; Roizman, 2011), it is unclear whether this phenotype is owing to the suppression of recruitment of the repressor complex to the virus genome or an indirect consequence of the de-repression of cellular gene expression. Further work in this area is clearly warranted, and in particular, further studies are required to determine whether the CoREST/REST repressor complex is recruited to the virus genome during neuronal latency.

## The nonuniformity of HSV latency

As has already been mentioned in this review, a striking feature of HSV latency concerns the nonuniformity of the latent state. Thus, viral DNA is not evenly distributed amongst latently infected neurons, and it is well established that the viral genome load can vary significantly, with the majority of cells harbouring 10–100 copies, whilst a smaller proportion can harbour > 1000 copies of virus DNA (Sawtell, 1997). As a consequence, analysis of latent virus DNA and in particular ChIP analyses are population weighted. The significance of this genome heterogeneity is unclear, although high latent genome copy number may be a predisposing factor for reactivation (Sawtell *et al*., 1998). The nonuniformity of latency also extends to the expression of LATs. Estimates of the number of neurones harbouring virus DNA by *in situ* PCR (Mehta *et al*., 1995; Maggioncalda *et al*., 1996) and laser capture microdissection (Chen *et al*., 2002; Wang *et al*., 2005a, b) indicate that LATs are transcribed in only a fraction (approximately 30%) of all latently infected cells. Whether the LAT promoter is ever active within cells negative by ISH is not clear, but data from our laboratory utilizing ROSA26R Cre reporter mice suggest that LAT expression occurs in a larger proportion of infected cells at some point during infection, indicative of dynamic expression of LATs during latency (Proença *et al*., 2008). Consistent with this view, data from LAT transgenic mice suggest that the LAT promoter can be expressed in the majority of sensory neurones (Gussow *et al*., 2006). Nevertheless, given evidence to date, precisely when the LATs are ‘needed’ and the role of LATs in the minority of LAT-expressing cells during latency remain pivotal questions.

To contemplate, HSV infection in terms of different neuronal populations necessitates the notion that latently infected neurons are not equivalent. Further still, the factors that pertain to this lack of equivalence are numerous and diverse. It has been shown that HSV-1 and HSV-2 preferentially establish latency and express LATs in different populations of sensory neurons identified by the expression of the cellular markers A5 and KH10, respectively (Margolis *et al*., 2007; Imai *et al*., 2009). Furthermore, whilst receptive to viral entry, A5^+^ neurons are intrinsically nonpermissive for productive infection and reactivation with HSV-1 but are able to support productive HSV-2 infection (Bertke *et al*., 2011). This intriguing observation indicates that the nonpermissiveness of A5^+^ neurones is largely specific for HSV-1. During mouse infection with HSV-1 mutants possessing HSV-2 LAT regions (and *vice versa*), these preferential sites swapped between viruses, defining the LAT as the viral determinant of this specificity (Margolis *et al*., 2007; Imai *et al*., 2009). These data demonstrate unequivocally that regulatory pathways significant to HSV infection act within individual neuronal subsets and that any concept of a homogenous population of neurons within a ganglion is a gross oversimplification. These data do not as of yet specifically address the functional bias upon replication exerted by these A5^+^ and KH10^+^ populations, but the authors do cite known differences in growth factor signalling between these cells. Specifically, A5^+^ neurons respond to NGF and KH10^+^ cells express receptors for glial cell-derived neurotrophic factor (GDNF). Given that approximately 50% of all neurones latently infected with HSV-1 are A5^+^ (Yang *et al*., 2000), it will be of particular interest to determine the characteristics of the remaining latent sites with respect to neuronal subtype and permissiveness to infection and reactivation competence. Work by others has shown that maintenance of quiescent HSV-1 infection in neuronal cultures *in vitro* requires NGF (Wilcox & Johnson, 1987) interaction with its high-affinity receptor, TrkA. Removal of NGF from neuronal culture resulted in recovery of productive infection, whilst only modest replication occurred after the removal of GDNF (Camarena *et al*., 2010). It would be of interest to explore whether A5^+^ neurons are rendered at all permissive to HSV-1 replication in the absence of NGF, whilst remaining viable in culture, as otherwise in the light of current data, this subtype appears to represent a ‘dead-end’ for productive HSV-1 replication. This is especially important given the interpretation of another area of nonuniformity during latent infection: the genome copy number per infected cell and the number of infected cells within a ganglion.

The implementation of CXA of viral DNA provided the first appreciation of virus genome copy number variability within latently infected cells (Sawtell, 1997). Utilizing CXA, it has been demonstrated that within a single neuron, latent HSV copy number can range from a single copy to > 1000 (Sawtell, 1997). At this time, it had already been hypothesized that latent cells bearing large HSV copy numbers may be predisposed towards reactivation relative to lower copy number cells and indeed CXA reveals that efficiently reactivating HSV-1 strains such as McKrae and 17syn+ display significantly larger average genome copy numbers compared to the poorly reactivating strain KOS (Sawtell *et al*., 1998). Interpreted one way, this observation could be at odds with the presence of ‘dead-end’ neuronal subtypes suggested by data mentioned previously. Any cells nonpermissive for replication could theoretically contain very high genome copy numbers if sequentially infected throughout primary disease (neurons directly innervating infection at the periphery, for example), so long as all productive replication was aborted in these cells. However, a mechanism for the onset of permissiveness of infection in such cells could render such neuronal populations highly effective reservoirs of reactivation. Another explanation for copy number variation that negates the requirement for superinfection of latently infected cells is that productive infections can be actively silenced at late stages of the HSV replication cycle. During ROSA26R reporter mouse infection, latently infected neuronal populations marked for ICP0 promoter activation represent one-third of the total infected cell reservoir (Proença *et al*., 2008). Follow-up data from our laboratory demonstrates the consistent presence of small numbers of neurones that have experienced early and late gene promoter activity (Proença *et al*., 2011). The implication of detecting latently infected cells marked for late promoter activity is that these cells are likely to have experienced and survived HSV genome replication before entering latency and thus may represent the small but constant population of high genome copy neurons detectable by CXA.

Finally, the form of heterochromatin that silences lytic gene expression during latency provides an additional layer of complexity. As previously mentioned, heterochromatin is divided into two classes: facultative and constitutive, with the former representing a ‘reversible’ mechanism of gene silencing, capable of reverting to euchromatin and the latter a more stable form of restriction associated with the centromeres and telomeres of mammalian DNA. In 2009, ChiP analyses by Cliffe *et al*. and Kwiatkowski *et al*. observed that lytic gene promoters could be found enriched for both facultative and constitutive chromatin PTMs within pooled samples of latently infected mouse tissue. These data raise the possibility that chromatin modification could be distinct not just between genomes in different cells, but also within the same cell (Bloom *et al*., 2010).

## Reactivation from latency

Lytic cycle viral gene products are not generally detected in latently infected neurons; therefore, reactivation from latency is thought to occur in the absence of pre-existing virus proteins by cellular mechanisms. The cellular factors involved in triggering reactivation of the latent genomes are currently unknown; however, several stimuli have been found to induce reactivation. In humans, exposure to UV light, emotional stress, fever, tissue damage and immune suppression is known inducers of reactivation. In cultured neurons, several stimuli have also been identified such as: NGF deprivation (Wilcox & Johnson, 1987, 1988; Wilcox *et al*., 1990), the histone deacetylase inhibitor Trichostatin A (Arthur *et al*., 2001), forskolin (Danaher *et al*., 1999), inducible cyclic AMP early repressor (Colgin *et al*., 2001), capsaicin (Hunsperger & Wilcox, 2003b), caspase-3 activator C2-ceramide (Hunsperger & Wilcox, 2003a, b), protein kinase C activation by phorbol myristate acetate (Smith *et al*., 1992), transient hyperthermia (Moriya *et al*., 1994) or addition of dexamethesone (Halford *et al*., 1996). In a recent study, Camarena and colleagues examined the signalling cascade responsible for viral reactivation upon NGF withdrawal. NGF is sensed by the TrkA receptor, which in the presence of NGF activates PI3-K (phosphatidylinositol 3-kinase) p110alfa catalytic subunit. This leads to the recruitment of 3-phosphoinositide-dependent protein kinase-1 (PDK1) to the plasma membrane and phosphorylation of serine/threonine kinase Akt (Camarena *et al*., 2010). The authors also found that epidermal growth factor (EGF) and GDNF growth factors although able to signal through the same pathways were not able to maintain HSV latency to a similar extent as NGF and concluded that this was probably due to their different abilities to sustain AKT phosphorylation (Camarena *et al*., 2010). It will be of interest to know the full signalling pathway that results in genome activation, specifically, which factors are involved in triggering the first viral transcripts. Understanding which viral genes are necessary for reactivation has been a long-lasting question in HSV research. The use of viral recombinants with mutations in different genes has been a common approach to define the role of individual gene products in reactivation; however, interpretation of such data is complicated because if a mutant virus has defects in lytic replication, this will not only impact on the efficiency of latency establishment but will also impact on full virus reactivation and infectious progeny production. Reactivation is classically scored by the formation of infectious particles from the latently infected tissue or cultures. Thus, if a mutant has defects in the lytic cycle, it will also impact on the formation of viral particles following the exit from latent infection. All essential genes are therefore necessary for reactivation but not all/any may be needed for reactivation at the molecular level as defined by the initial events that lead to the switch between latency and the entry into productive cycle.

Several systems have been used to study HSV-1 reactivation from latency. The simplest and probably the most artificial is the *in vitro* quiescent model where viral genomes deficient for IE gene expression become tightly repressed 24–48 h postinfection (Preston *et al*., 1997; Samaniego *et al*., 1998; Coleman *et al*., 2008). In this system, once tight repression is established, the only HSV-1 protein able to de-repress the quiescent genomes is ICP0.

Latently, infected neuronal cultures can be reactivated by several other stimuli including other HSV proteins: such as VP16 or ICP4, which can be delivered by superinfection with adenovectors (Halford *et al*., 2001). Neuronal latency is therefore characterized as having a more relaxed state of repression, in at least a subpopulation of latently infected cells (Arthur *et al*., 2001; Terry-Allison *et al*., 2007). However, neuronal culture latency is generally established following an unnatural block in the viral lytic cascade of gene expression through the use of replication defective virus mutants or the inclusion of inhibitors of viral DNA replication. Such experimental methodologies therefore result in latency being established in cells that may otherwise have supported lytic cycle replication. It is currently unclear whether the ‘repressed’ state observed in such latently infected neurones resembles natural latency.

*In vivo*, it seems that only a small proportion of latently infected cells are competent for reactivation even following stimuli that affects the whole ganglion. A good example is the *in vivo* hyperthermia induced reactivation model described by Sawtell and Thompson where, on average, only 1–3 neurons per TG (approximately 1 in 2700 latently infected cells) reactivate and are antigen-positive 22-h poststimulus (Sawtell & Thompson, 1992; Thompson & Sawtell, 2010). In a similar fashion, cultures of dissociated ganglia from latently infected mice also display a low frequency of spontaneous reactivation (Halford *et al*., 1996).

In humans, it is clear that spontaneous reactivation leading to the shedding of virus in the genital mucosa occurs at high frequency (reviewed by Koelle & Corey, 2008; Schiffer & Corey, 2009). Thus, the frequency of asymptomatic genital HSV-2 shedding averages 25% of days, and such reactivation events have a rapid onset and are cleared within 12 h (Wald *et al*., 2000; Mark *et al*., 2008). Thus, in contrast to the situation in mouse infection models, latent neuronal infection in humans is less tightly controlled and the resultant high frequency of reactivation is a major factor in human-to-human transmission (Koelle & Corey, 2008).

Of critical importance in understanding the mechanism of virus reactivation is the responsiveness of viral promoters to triggers of virus reactivation and the identity of viral gene(s) responsible for the initiation of the lytic gene expression and exit from latency. ICP0 has always been regarded as the most likely candidate owing to its ability to target ND10 domains for degradation and its capacity to act as a promiscuous activator of gene expression (reviewed by Everett, 2000). This role is strongly supported by the fact that in the *in vitro*, nonneuronal quiescence model, ICP0 is the only HSV protein able to reverse the quiescent state (Harris *et al*., 1989; Stow & Stow, 1989; Preston *et al*., 1997; Samaniego *et al*., 1998; Coleman *et al*., 2008; Ferenczy *et al*., 2011). Furthermore, *in vivo* ICP0 null mutants do not efficiently reactivate following explant culture of latently infected ganglia (Cai *et al*., 1993; Halford & Schaffer, 2001). A key question is whether the deficits in reactivation observed for ICP0 mutants represent a failure to initiate exit from latency or failure to enter productive cycle replication following the initiation of reactivation. Studies by Thompson & Sawtell (2006) have provided evidence for the latter theory that the molecular trigger responsible for ‘primary’ reactivation from latency is not ICP0. In this study, similar latent loads were achieved in mice infected with an ICP0 mutant or WT virus and 22 h following the application of hyperthermic stress, a similar number of antigen-positive cells were found to exit from latency. Thus, following the application of an *in vivo* reactivation stimulus, ICP0 appears to play no important role in the initiation of reactivation from latency but is required for progression to virus production following exit from latency.

A recent publication by Thompson and Sawtell (Thompson *et al*., 2009) identified VP16 as the molecular trigger for reactivation following hyperthermic stress. This was an unexpected finding given that VP16 is classically defined as a late gene product and therefore optimally expressed only after the onset of virus DNA replication. Given its late temporal class, it seemed unlikely that this potent transactivator would have a pivotal role in the earliest steps of virus reactivation; yet, the VP16 promoter was found to have a distinct regulation in neurons, and *de novo* synthesis of VP16 was found to be necessary for efficient initiation of the viral lytic cycle in neurons, both during reactivation from latency but also during the acute phase of infection (Thompson *et al*., 2009). Of particular importance will be to determine the responsive elements of the VP16 promoter and identification of cellular transcription factors that mediate promoter activation in response to reactivation triggers. If VP16 is the trigger for reactivation, it would be fair to assume that its co-factors Oct-1 and HCF-1 would also be present in order to efficiently induce IE gene expression. In fact, upon ganglionic explant, the most commonly used reactivation stimulus, Oct-1 expression is induced in sensory neurons (Valyi-Nagy *et al*., 1991). Furthermore, HCF-1 has been shown to relocalize from the Golgi to the nucleus (Kristie *et al*., 1999; Kolb & Kristie, 2008) in up to half the neurons in explanted TGs and associates with viral IE promoters as early as 1-h postganglionic explant (Whitlow & Kristie, 2009). Recent data have also shown that HCF-1 recruits the lysine-specific demethylase (LSD1) to virus IE promoters to reverse repressive histone methylation marks to facilitate both lytic cycle virus gene expression and reactivation from latency (Liang *et al*., 2009). This new described function of VP16 correlates with its large-scale chromatin remodelling features (Tumbar *et al*., 1999) and potent transcriptional activator functions (reviewed in detail by Kutluay & Triezenberg, 2009). Experimental data using adenovirus vectors confirm the ability of VP16 to reactivate latent genomes and that this is dependent on its acidic transactivator domain (Halford *et al*., 2001). Neuronal cultures systems have shown that superinfection of latently infected cells with an IE null mutant can result in reactivation possibly via delivery of VP16 although less efficiently than with a similar mutant able to express ICP0 (Terry-Allison *et al*., 2007). Furthermore, a study by Jacob *et al*. where mutants were tested for their ability to reactivate following hyperthermia, forskolin or trichostatin A (TSA) treatments from latently infected PC12 cells revealed that only mutants lacking functional VP16 failed to reactivate (Miller *et al*., 2006).

**Fig 3:**
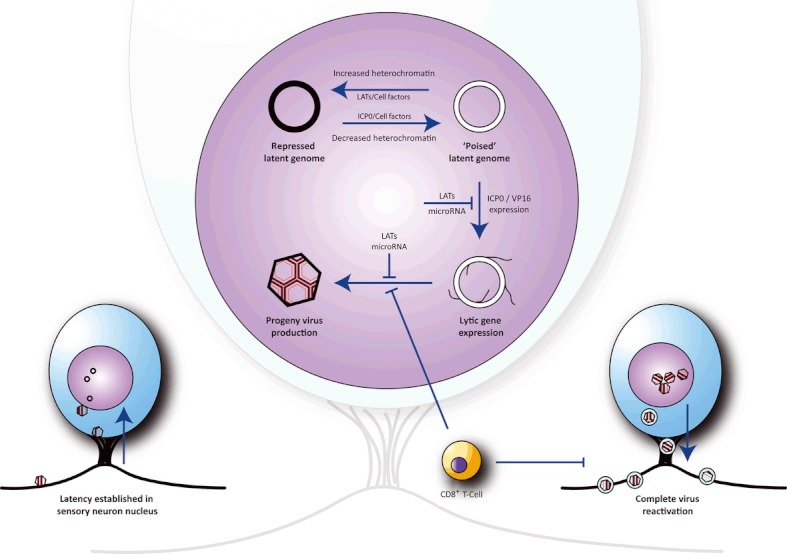
The balance between latency and productive infection/reactivation is policed by cellular and viral factors. HSV latency is established in sensory neurons innervating the sit of primary infection. During the establishment of latency, within the cell nucleus, circularized viral DNA becomes associated with heterochromatin resulting in the repression of lytic cycle gene expression. Periodically, virus reactivation and exit from latency can occur as a consequence of changes in neuronal cell physiology in response to stress. Reversal of repressive histone modifications allows transcriptional activation of one or more viral gene products responsible for initiating virus replication. Accumulation of the LATs and cognate microRNAs during latency may facilitate establishment of latency and regulate reactivation by blocking translation of the viral IE genes ICP0 and ICP4. Ganglion-resident CD8^+^ T cells may actively halt reactivation through noncytolytic control mechanisms. Occasionally, reactivating cells escape cellular, virus and immune mechanisms of control leading to the completion of the virus life cycle, and production of progeny virus that is then transported to the periphery resulting in either asymptomatic or symptomatic infection that can lead to transmission of reactivated virus to immunologically naïve hosts.

Reactivation from latency is a critical phase of HSV life cycle; therefore, it is not surprising that it is highly regulated. It seems that only a small proportion of latently infected neurones are able to respond to a given reactivation stimulus at any given time. The responsiveness of latent virus genomes may be related to the nonuniform nature of the latent state. Thus, multiple factors such as latent DNA copy number, level of LAT expression, neuronal type, degree of genome repression and CD8^+^ T cell immunosurveillance may influence both the efficiency and mechanism by which reactivation can occur (Fig. 3). Given this knowledge, it maybe difficult to arrive at a simple model for the molecular basis of HSV latency and reactivation. An effort in understanding the nature and biological significance of the heterogeneity of latency is therefore warranted.

## Statement

Re-use of this article is permitted in accordance with the Terms and Conditions set out at http://wileyonlinelibrary.com/onlineopen#OnlineOpen_Terms
